# Supplementary material for: Towards Embedded Computation with Building Materials
Source: Materials (Basel). 2021 Mar 31;14(7):1724. doi: 10.3390/ma14071724 (PMC8038044; doi:10.3390/ma14071724)
Supplement: Supplementary file 1 [file materials-14-01724-s001.pdf]

Electronic supplementary information

Towards embedded computation with building materials

Dawid Przyczyna[ab]*, Maciej Suchecki[ab], Andrew Adamatzky[c], Konrad Szaciłowski[a]*

[a]AGH University of Science and Technology, Academic Centre for Materials and Nanotechnology, al. Mickiewicza 30, 30-059 Kraków, Poland

[b]AGH University of Science and Technology, Faculty of Physics and Applied Computer Science, al. Mickiewicza 30, 30-059 Kraków, Poland

[c]University of the West of England, Unconventional Computing Lab, Department of Computer Science and Creative Technologies, Bristol BS16 1QY, United Kingdom

*Corresponding authors: dawidp@agh.edu.pl, szacilow@agh.edu.pl

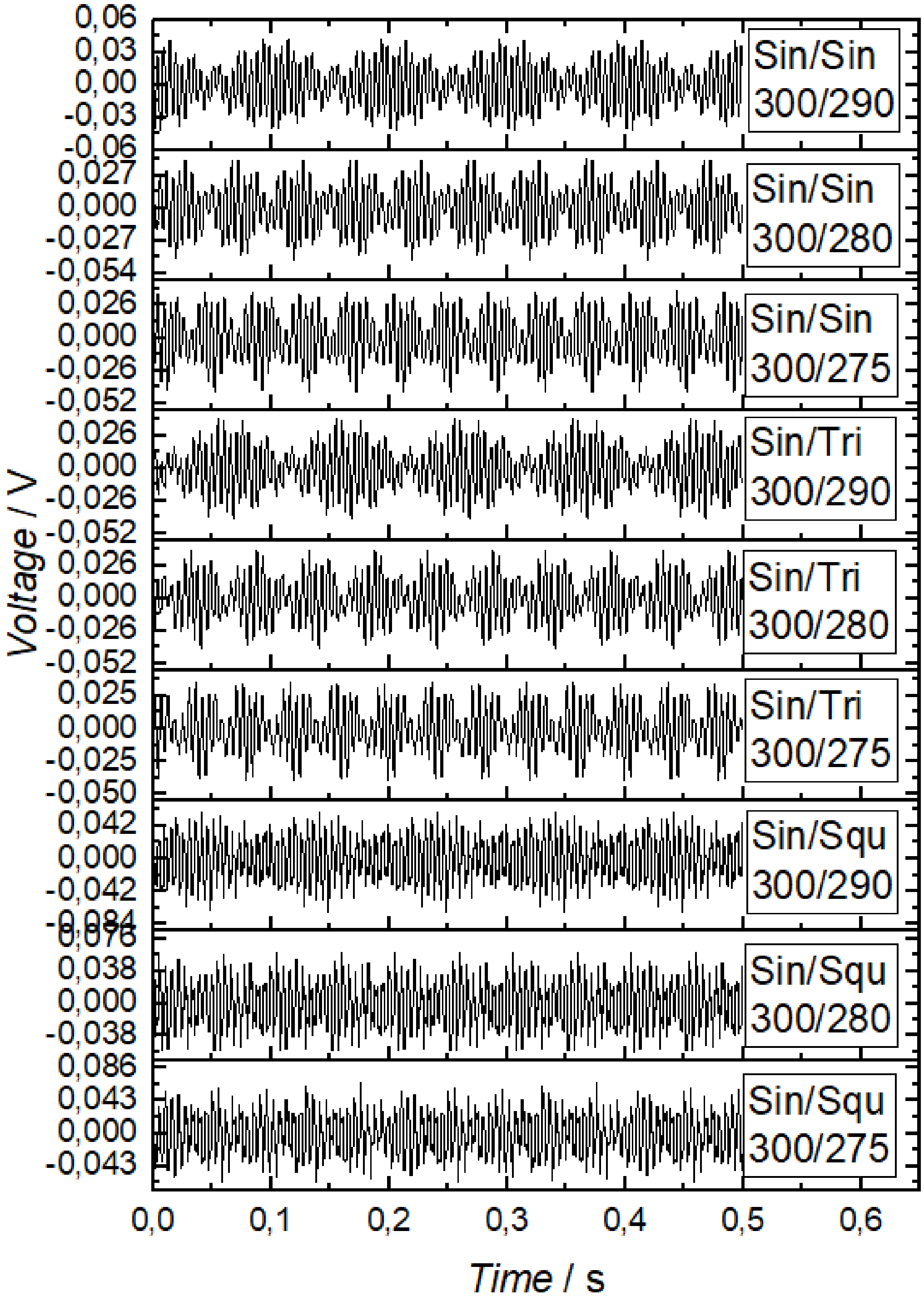

Figure S1. Registered timeseries for undoped concrete sample (Sin – sinusoidal, tri – triangular, squ – square wave form). Frequencies are given in Hz.

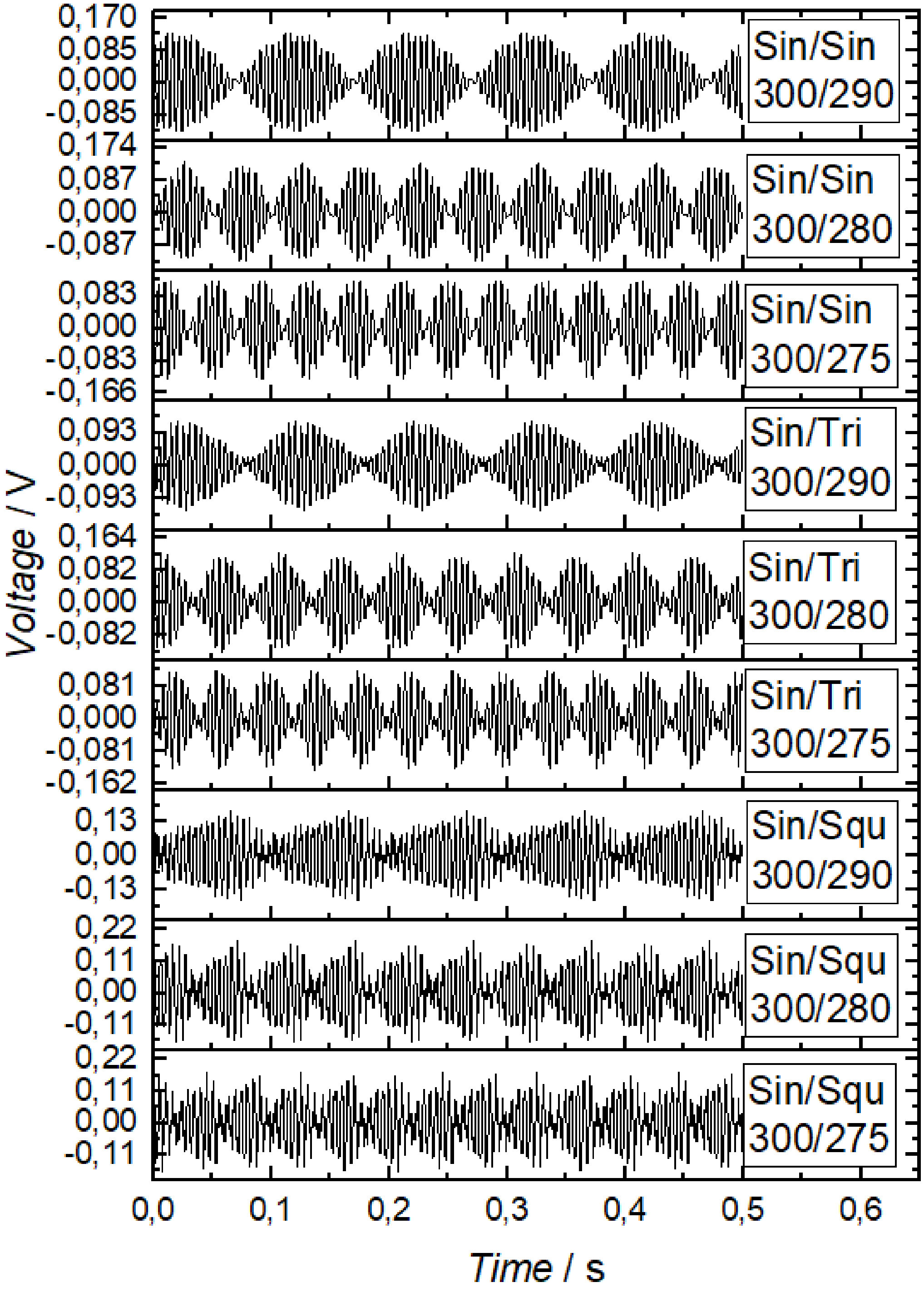

Figure S2. Registered timeseries for doped concrete sample (Sin – sinusoidal, tri – triangular, squ – square wave form). Frequencies are given in Hz.

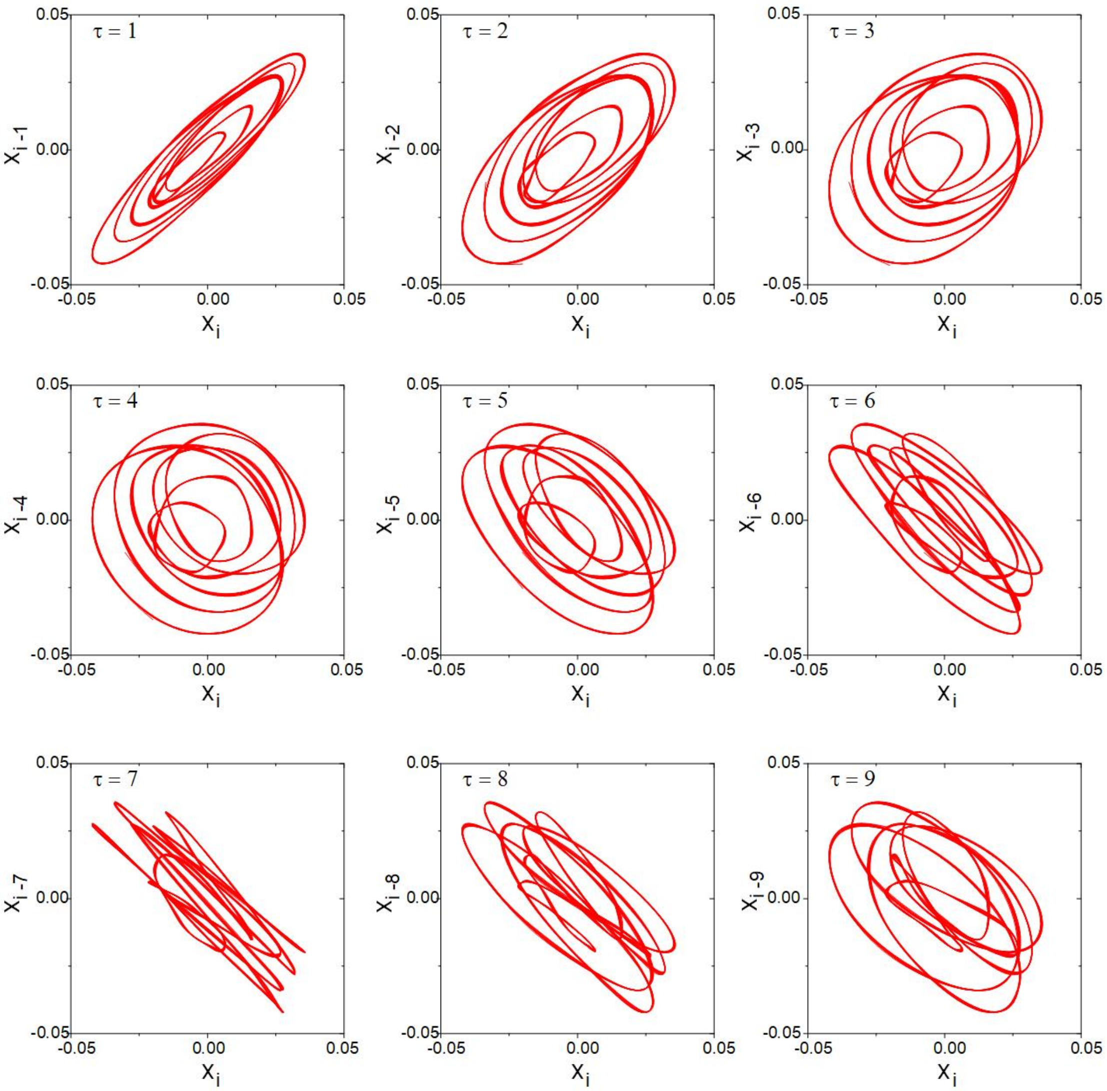

Figure S3. 2D projections of trajectories (return plots) of the signal obtained for two sine waves (275 and 300 Hz) in undoped concrete. The case of $\tau = 4$ does not show any diagonal stretching.

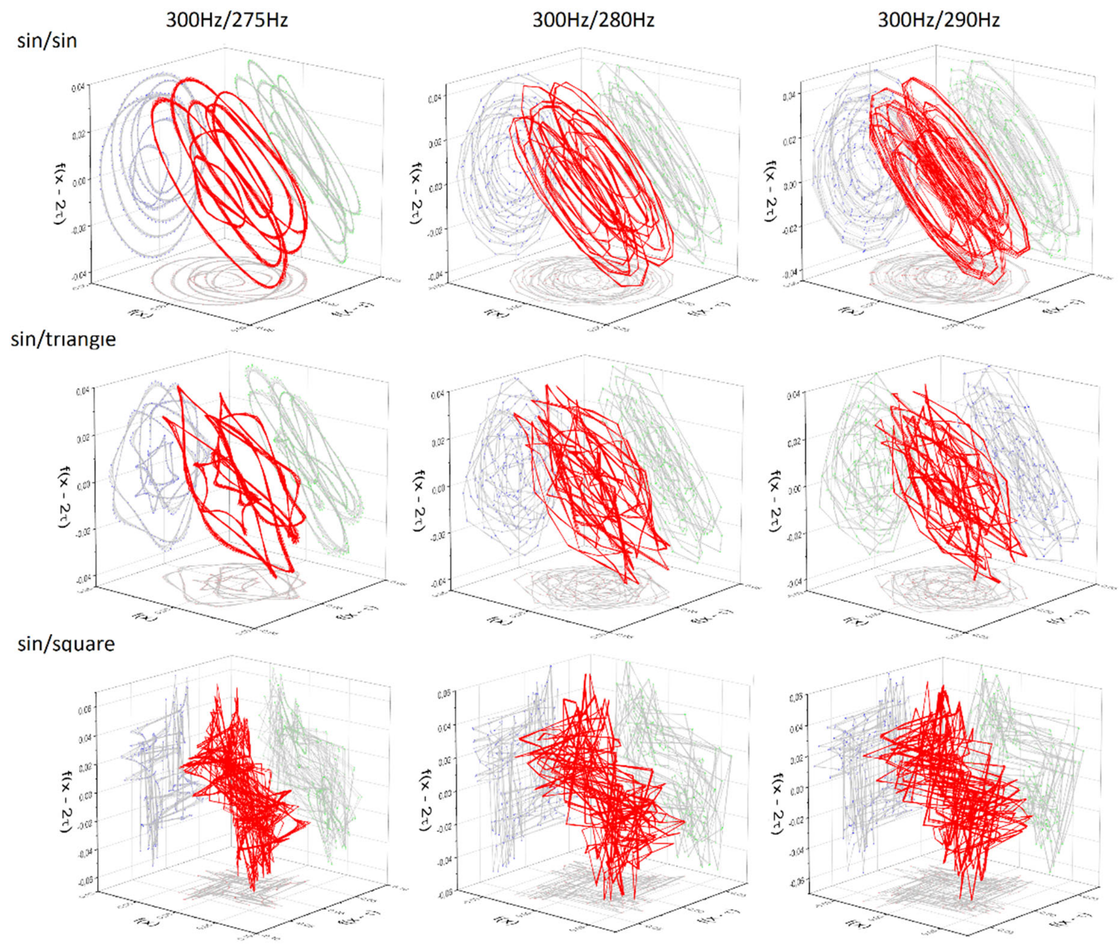

Figure S4. Embedded time-delay trajectories of time series recorded for un-doped concrete sample for various input waveforms and frequencies, constructed with time delay τ = 4. It should be noted that the f(x) nv f(x-τ) projections are free from diagonal distortions, which supports the evaluated τ value.

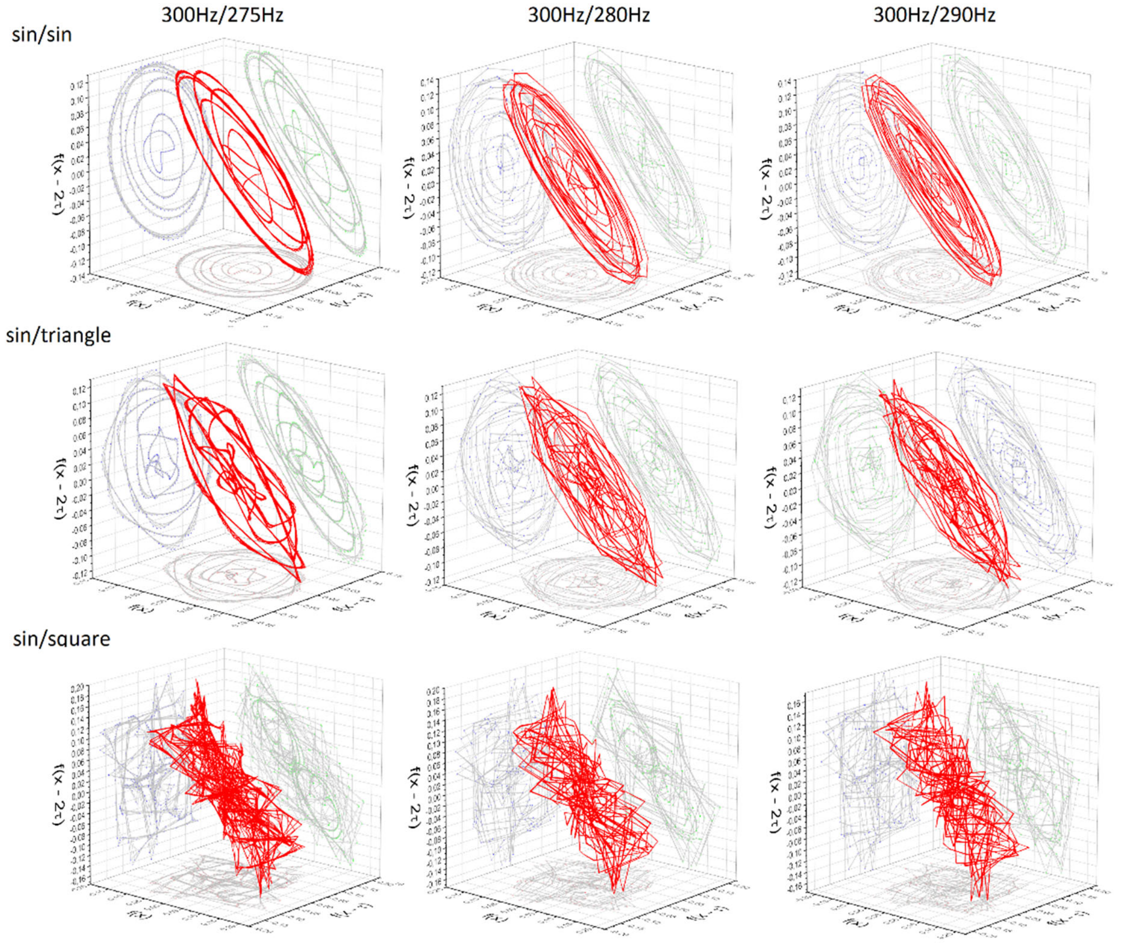

Figure S5. Embedded time-delay trajectories of time series recorded for doped sample (10% SM) for various input waveforms and frequencies, constructed with time delay τ = 4. It should be noted that the f(x) nv f(x-τ) projections are free from diagonal distortions, which supports the evaluated τ value.
